# A Remote Medication Monitoring System for Chronic Heart Failure Patients to Reduce Readmissions: A Two-Arm Randomized Pilot Study

**DOI:** 10.2196/jmir.5256

**Published:** 2016-05-06

**Authors:** Timothy M Hale, Kamal Jethwani, Manjinder Singh Kandola, Fidencio Saldana, Joseph C Kvedar

**Affiliations:** ^1^ Partners Healthcare Connected Health Boston, MA United States; ^2^ Massachusetts General Hospital Boston, MA United States; ^3^ Harvard Medical School Boston, MA United States; ^4^ Brigham and Women's Hospital Internal Medicine Boston, MA United States

**Keywords:** heart failure, telemonitoring, telehealth, self-management, self-care, complex medication regimens, medication management, medication adherence, hospitalization length of stay, ED visits

## Abstract

**Background:**

Heart failure (HF) is a chronic condition affecting nearly 5.7 million Americans and is a leading cause of morbidity and mortality. With an aging population, the cost associated with managing HF is expected to more than double from US $31 billion in 2012 to US $70 billion by 2030. Readmission rates for HF patients are high—25% are readmitted at 30 days and nearly 50% at 6 months. Low medication adherence contributes to poor HF management and higher readmission rates. Remote telehealth monitoring programs aimed at improved medication management and adherence may improve HF management and reduce readmissions.

**Objective:**

The primary goal of this randomized controlled pilot study is to compare the MedSentry remote medication monitoring system versus usual care in older HF adult patients who recently completed a HF telemonitoring program. We hypothesized that remote medication monitoring would be associated with fewer unplanned hospitalizations and emergency department (ED) visits, increased medication adherence, and improved health-related quality of life (HRQoL) compared to usual care.

**Methods:**

Participants were randomized to usual care or use of the remote medication monitoring system for 90 days. Twenty-nine participants were enrolled and the final analytic sample consisted of 25 participants. Participants completed questionnaires at enrollment and closeout to gather data on medication adherence, health status, and HRQoL. Electronic medical records were reviewed for data on baseline classification of heart function and the number of unplanned hospitalizations and ED visits during the study period.

**Results:**

Use of the medication monitoring system was associated with an 80% reduction in the risk of all-cause hospitalization and a significant decrease in the number of all-cause hospitalization length of stay in the intervention arm compared to usual care. Objective device data indicated high adherence rates (95%-99%) among intervention group participants despite finding no significant difference in self-reported adherence between study arms. The intervention group had poorer heart function and HRQoL at baseline, and HRQoL declined significantly in the intervention group compared to controls.

**Conclusions:**

The MedSentry medication monitoring system is a promising technology that merits continued development and evaluation. The MedSentry medication monitoring system may be useful both as a standalone system for patients with complex medication regimens or used to complement existing HF telemonitoring interventions. We found significant reductions in risk of all-cause hospitalization and the number of all-cause length of stay in the intervention group compared to controls. Although HRQoL deteriorated significantly in the intervention group, this may have been due to the poorer HF-functioning at baseline in the intervention group compared to controls. Telehealth medication adherence technologies, such as the MedSentry medication monitoring system, are a promising method to improve patient self-management,the quality of patient care, and reduce health care utilization and expenditure for patients with HF and other chronic diseases that require complex medication regimens.

**Trial Registration:**

ClinicalTrials.gov NCT01814696; https://clinicaltrials.gov/ct2/show/study/NCT01814696 (Archived by WebCite® at http://www.webcitation.org/6giqAVhno)

## Introduction

Heart failure (HF) affects nearly 5.7 million Americans today, contributing to 1 in 9 deaths in 2011 [1]. Furthermore, with an aging population and advancements in survival following acute cardiac injury, such as myocardial infarction, the burden of HF is expected to increase by 46% in prevalence between 2012 to 2030, resulting in approximately 8.5 million adults with HF [1,2].

In addition to its effect on morbidity and mortality, the management of HF is incredibly expensive, with aggregate costs projected to grow from US $31 billion to US $70 billion from 2012 to 2030 [2]. Heart failure remains the most frequent cause of hospitalization in patients older than 65 years [3]. Moreover, discharged patients often have subsequent complications, with 15-day readmission rates estimated at approximately 13% and 30-day readmission rates at approximately 25%; nearly half of all patients are readmitted at 6 months [4]. Unfortunately, despite efforts to improve the quality of HF care, readmission rates have not improved [4].

### Heart Failure Telehealth Interventions

The burden of HF has created a sense of urgency to develop, optimize, and evaluate programs that can enable more effective transitions to self-management in patients with HF [5]. A range of new technologies (eg, high-speed Internet, wireless connections, small inexpensive sensors) enable the creation and implementation of new health care intervention strategies to improve patient self-management and achieve both more efficient care and better health outcomes [6]. Telehealth is “the application of technologies to help patients manage their own illnesses through improved self-care and access to education and support systems” [6]. Telemedicine, a closely related term, is the use of technology to deliver care at a distance to improve access, quality, and cost of care [6]. Telehealth and telemedicine programs can allow physicians and nurses to reduce face-to-face time with patients while maintaining the quality and quantity of care [7,8].

To date, most HF telehealth interventions use noninvasive remote monitoring (ie, telemonitoring) of patients’ body weight and other measures, including blood pressure, heart rate, blood oxygen saturation, and patient-reported symptoms [9]. In the advent of signs of deteriorating health, nurses communicate with physicians and patients to coordinate changes in treatment and/or schedule a visit to the clinic [9]. Research has shown that patients with chronic HF who range in age between 55 and 85 years feel confident and comfortable using telemonitoring devices [10], which allow them to experience the benefits of independence, the ability to measure and monitor their vitals, and to better manage their disease [11].

Meta-analyses of HF telemonitoring trials have been encouraging [12]. Clark et al [13] found telemonitoring programs reduced HF-related hospitalization rates by 21% and all-cause mortality by 20%. They also found significant improvement in health-related quality of life (HRQoL) in three of six studies. A meta-analysis of 13 studies found a reduction in HF-related hospitalization and all-cause mortality over 3 to 15 months follow-up [14]. Klersy et al [15] conducted a meta-analysis of 20 randomized controlled trials (RCTs) and 12 cohort studies with a median follow-up duration of 6 months in RCTs and 12 months in cohort studies. They found that telemonitoring programs were associated with lower mortality and fewer HF-related and all-cause hospitalizations. Although a large follow-up RCT conducted by Chaudhry et al [16] failed to replicate these findings, the most recent meta-analysis of HF telemonitoring studies conducted in 2015 found improved outcomes [17] and a number of recent HF telemonitoring trials suggest the possibility and promise of these technologies to improve care and clinical outcomes [18-26].

Although many HF telemonitoring interventions have been found to be useful immediately following hospitalization, their long-term impacts remain to be fully characterized [9] and the benefits may decrease over time. For example, Clarke et al [14] found that the reduced risk of hospitalization seen at 12 months postdischarge was no longer significant at 15 months. Agboola et al [18] observed a similar pattern with decreased hospitalizations at 30 days and at the end of the 4-month program. However, there was no observed reduction in hospitalizations at 12 months. Therefore, there is a need to find alternative telemonitoring strategies and devices that are effective in helping HF patients to self-manage their medical condition on a long-term basis.

### Medication Adherence

One key component of HF patient self-management is medication adherence. Poor medication adherence contributes to poor quality of life [27] and increased risk of mortality and morbidity [28], hospitalization [28,29], and high health care costs [2] among HF patients. Adherence ranging from 2% to 90% has been reported [30,31] with a typical adherence of approximately 40% to 60% [28,32]. It is estimated that poor adherence among HF patients contributes to approximately US $300 billion in health care costs each year [33].

There is widespread agreement that efforts to improve medication adherence and patient self-care are needed to improve HF patients’ quality of life and reduce the risk of hospitalization [34,35]. However, achieving high medication adherence is difficult in this population for several reasons. First, adequate adherence for HF patients may be higher than what is needed to manage other medical conditions. Adequate adherence is often defined as 80% or greater, but Wu et al [36] found that daily dose adherence of 88% or greater was required to achieve longer time to first emergency department (ED) visit and decreases in hospitalizations and mortality among HF patients. Second, HF patients are often prescribed a complex medication regimen consisting of multiple medications to achieve symptom control, reduce morbidity and mortality, and to improve quality of life [37,38]. Finally, cognitive deficits and memory problems, which are more common among older adults with HF [39,40], make it difficult for patients to remember to take their medications [29]. Riegal and Knafl [29] found that impaired cognition was an important factor interacting with poor adherence and recommend using devices to remind patients to take their medications. Therefore, a solution is needed to help HF patients better manage their medications and remember to take them as prescribed long after traditional telemonitoring interventions end.

### Medication Adherence Telehealth Interventions

An electronic remote medication monitoring system could prove efficacious in aiding HF patients to adhere to their complicated medication regimen. Evidence suggests that patient-focused adherence interventions are more effective in improving adherence rates than efforts directed at health care provider behaviors [32]. In fact, many recent remote monitoring interventions have sought to specifically improve medication adherence through diverse means, such as increased patient education and health literacy, provider and pharmacist consultation, phone-based adherence assessments and positive behavior encouragement, and electronic reminders. However, many of these are early phase trials and results are so far met with cautious optimism. Although many suggest usability and patient satisfaction, as well as improved adherence measures, their ultimate effect on health outcomes remains largely unmeasured and requires further evaluation [32,41,42].

### Study Goals

The primary goal of this randomized controlled pilot study is to compare the MedSentry medication monitoring system versus usual care in older HF adults in patients who recently completed a HF telemonitoring program. The MedSentry medication monitoring system is a novel technology consisting of two parts: (1) a remotely monitored electronic pillbox that alerts people when it is time to take their medications and (2) a monitoring center with advisors who contact patients and caregivers when medications are not taken. We hypothesized that remote medication monitoring would be associated with fewer unplanned hospitalizations and ED visits, increased medication adherence, and improved HRQoL versus usual care. We also examined user satisfaction and usability with the MedSentry medication monitoring system among participants in the intervention arm.

## Methods

### Study Design

This pilot study was conducted as a RCT. Participants randomized to the intervention group used the MedSentry medication monitoring system for medication management. Participants randomized to the control group continued to use their usual medication reminder method. The intervention period was 90 days. Participants were given the option to enroll in a second 90-day period in which participants in the control group used the MedSentry medication monitoring system and participants in the intervention arm returned to their usual medication reminder method. The results reported in this paper examine only the first 90-day intervention period. The initial goal was to enroll a total of 70 participants with 35 randomized to each of the two study arms. However, due to slow enrollment, the study was ended early with 29 participants who completed enrollment and randomization.

### Study Participants

Participants were recruited from a list of HF patients who had successfully completed a HF telemonitoring program at Massachusetts General Hospital (MGH) or Brigham and Women’s Hospital (BWH) and had been hospitalized in the previous 24 months. Patient lists were reviewed by study staff to identify potential participants based on other inclusion/exclusion criteria and, if met, were approved by the MGH- or BWH-affiliated physician for participation in the study.

The inclusion criteria for this study were (1) take at least three and no more than 10 different daily medications, (2) take medications no more than four specified times each day (ie, morning, afternoon, early evening, bedtime), (3) able to sort and manage their own medications, (4) have a telephone or cell phone, (5) live in the greater Boston area, and (6) speak, read, and write English. Patients were excluded if they met any of the following: (1) vision or hearing impaired (ie, unable to hear an alarm similar to a clock alarm or oven alarm), (2) dementia or other conditions precluding the participant from providing informed consent, (3) awaiting revascularization, cardiac resynchronization, or heart transplant, and (4) terminal illness.

Eligible patients were screened by phone to confirm eligibility and be informed of the study. Due to the difficulty in scheduling the device installation, patients who agreed to participate were randomized during the screening phone call. Participants assigned to the control group provided consent over the phone and were mailed a copy of the consent form and enrollment questionnaire to complete. Participants assigned to the intervention group were scheduled for a study visit in which they completed the informed consent process and were trained in the use of the MedSentry device. The Partners HealthCare Institutional Review Board approved all study procedures.

### The MedSentry Medication Management System

The MedSentry medication monitoring system consists of two parts: (1) a remotely monitored electronic device (“device”) that alerts participants when it is time to take their medications and (2) a monitoring center with advisors who contact participants and caregivers when medications are not taken. The device is installed in the participant’s home and data are transmitted to the monitoring center via the Internet.

The device is approximately the size of a small microwave oven. The top of the device consists of a series of small, removable bins arranged in a 7 by 4 configuration (seven days of the week and four medication times per day). A lid on the top of each bin detects when a bin is opened. The bottom of each bin is clear plastic. Cameras located under the bins transmit an image of the contents to the monitoring center. During the installation, participants were trained in how to refill the device with the medications they were prescribed for each time of day. The monitoring center was provided a medication list and an image of the correctly loaded medications for reference.

The MedSentry medication monitoring system uses several methods to ensure participants take their medications as prescribed. First, the device provides a visual cue (blue lights around a bin) and an audio alarm to alert a participant when it is time to take their medication. If a dose is not taken within 30 minutes, an advisor at the monitoring center calls the participant. After three attempts over a 45-minute time span to contact the participant, a voice message is left and a call is placed to an optional caregiver who has agreed to be contacted and to follow up with the participant.

Participants were responsible for refilling the device and communicating medication changes to the monitoring center. Each week, the monitoring center advisor called to remind participants to refill the device. When participants refilled the device, a new image was transmitted and compared at the monitoring center. Participants were contacted by phone to correct refill errors. When there was a change in medications, participants used the “call request button” on the front of the device to notify the monitoring center of changes. The monitoring center verified that the pill tray was filled correctly and updated the medication list and reference image. Monthly medication adherence reports were sent to the participant’s physician and caregiver, if requested.

### Data Collection and Outcome Measures

Participants in both study arms completed enrollment and closeout questionnaires (Multimedia Appendix 1). Participants in the control arm completed and returned questionnaires by mail. Participants in the intervention arm completed questionnaires at the installation visit and at closeout when the device was removed from their home. The enrollment questionnaire collected data on demographics and baseline technology use. The closeout questionnaire for the intervention arm participants included questions to assess satisfaction and usefulness of the MedSentry device and monitoring services and their willingness to recommend the device.

The enrollment and closeout questionnaires contained items to assess changes in medication adherence, health, and HRQoL. Medication adherence was assessed using a self-reported measure and data collected by the device. Self-reported adherence was assessed using a single question from the Medical Outcomes Study (MOS) [43,44]. Participants were asked, “How often did you take your medications as prescribed (on time without skipping doses) during the past 4 weeks?” A dichotomous measure was created to indicate “nonadherent” (none, a little, or some of the time) or “adherent” (most or all the time). A direct measure of medication adherence was derived using data from the MedSentry device and monitoring center logs. Adherence for each 30-day period was calculated using data on the number of pills taken divided by the number of pills prescribed. If medications were not taken within 1 hour of the prescribed time, they were coded as “missed” and coded as “taken” if confirmed by an outreach phone call from the monitoring center.

Health was assessed using a single item on self-reported general health status (1=poor to 5=excellent). Depression was assessed using the 8-item Patient Health Questionnaire (PHQ-8). Each of the PHQ-8 items is scored from 0 to 3 to generate a total score from 0 to 24. Generally accepted cut points for depression severity are 5-9=mild, 10-14=moderate, 15-19=moderately severe, and 20-24=severe [45]. The PHQ-8 has been found to be a valid and reliable measure of depression among HF patients [46]. To assess baseline heart function the New York Heart Association (NYHA) Functional Classification was extracted from participants’ electronic medical records for the clinic visit nearest the study enrollment date. Heart functioning is classified as one of four levels, ranging from Class I=“no symptoms with ordinary activity” to Class IV=“unable to carry out any physical activity without discomfort; symptoms of cardiac insufficiency may be present even at rest.” When no classification was recorded in the electronic medical record (EMR), the study research nurse created a classification based on the clinical notes recorded nearest the study enrollment date.

Health-related quality of life was measured using the Minnesota Living with Heart Failure Questionnaire (MLHFQ) [47]. The questionnaire consists of 21 items that assess the impact of HF and HF treatment on key physical, emotional, and social dimensions of a patient’s life during the past four weeks. Responses are coded from 0=does not apply and 1=very little to 5=very much. In addition to a total summary score, a physical subscore and emotional subscore can be created [47,48]. The instrument has good construct validity and test-retest reliability [47,49].

Data on hospitalization and ED visits came from two sources. The primary source was the participants’ EMR. The second source was a series of questions on the closeout questionnaire about the type and timing of hospitalization and ED visits during the 90-day study period. Our analysis focused on unplanned hospitalizations; therefore, planned hospitalizations were excluded from our calculations of hospital visits and days hospitalized.

### Data Analysis

Baseline characteristics were summarized and compared for both study arms using percentages for categorical variables and means and standard deviations for continuous variables. Comparisons on outcome measures between intervention and control arms at the close of the study were conducted using unpaired *t* tests, unpaired proportion test, and the Fisher exact test. For skewed data (ie, ED visits and days hospitalized), Wilcoxon rank sum tests were conducted. Descriptive statistics were used to summarize results on usability and satisfaction with the MedSentry medication monitoring system. Data analysis was performed with Stata 14 with an alpha of .05 set a priori *.*

## Results

### Study Flow

**Figure 1:**
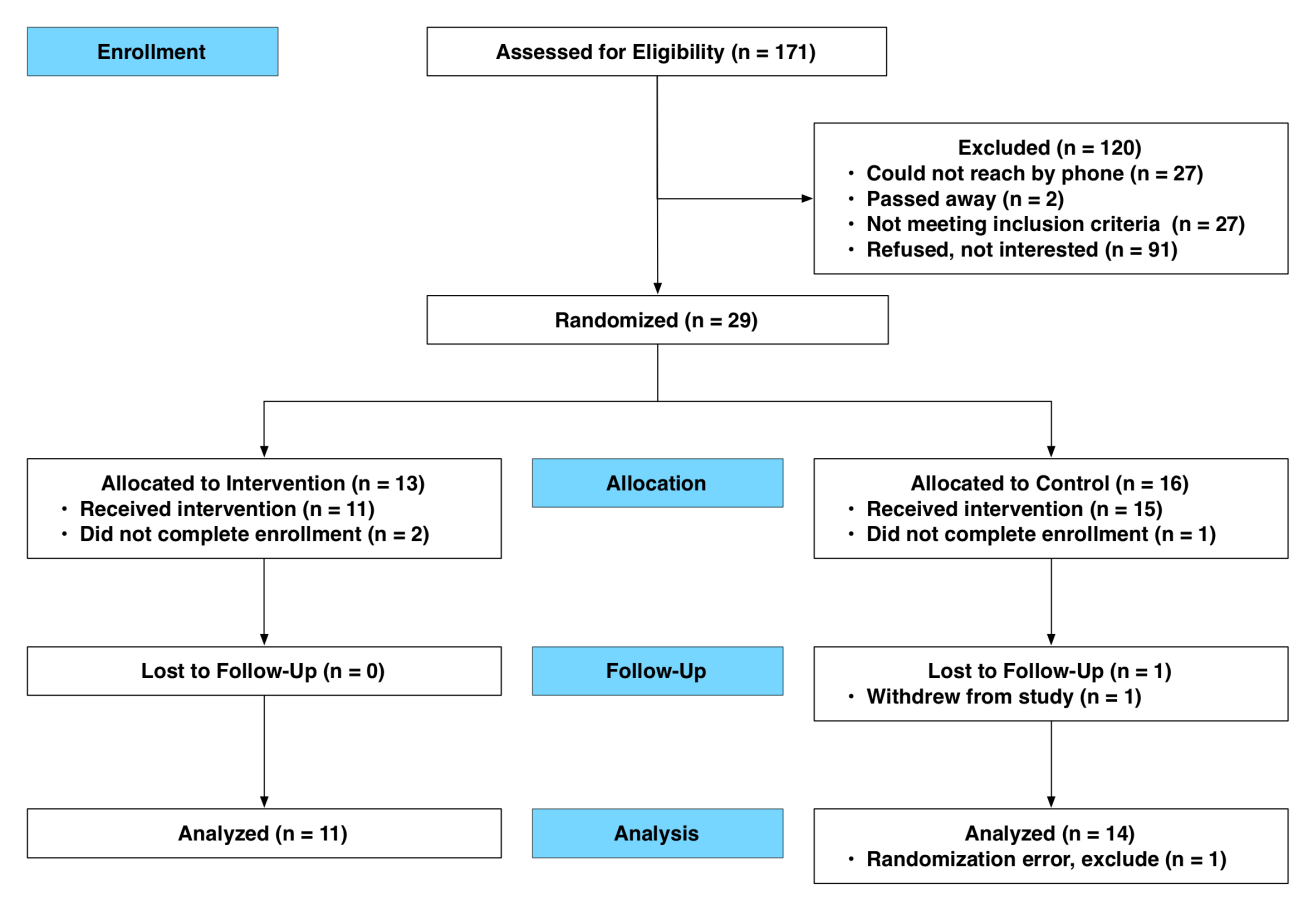
Participant enrollment and inclusion.

Of the 171 patients assessed for eligibility, 29 were randomized (see Figure 1). Thirteen participants were randomized to the intervention arm, two participants did not complete the enrollment process, and 11 completed the study. In total, 16 participants were randomized to the control group; however, one did not complete the enrollment process and one was mistakenly randomized to the control group and was excluded from analysis. Of the 14 enrolled participants, one withdrew leaving 13 participants who completed the study. Conducted as an intention-to-treat analysis, we included the participant who withdrew from the study in the analysis of hospitalizations and ED visits.

### Baseline Characteristics

Descriptive statistics for demographics and baseline characteristics are presented in Table 1. The mean age of participants was 71.7 years (SD 11.2) and 64% (16/25) were male. Participants were predominantly white and had one or more years of college education. Overall, 44% (11/25) were married, 40% (10/25) lived alone in the household, 64% (16/25) had used the Internet, and 72% (18/25) had a cellphone. There were no statistically significant differences in these characteristics between participants in the intervention and control arms. Of the 25 participants, 18 (72%) reported they currently used a medication reminder tool or method. Seventy-two percent (18/25) of participants were categorized as “adherent” based on self-reported adherence.

**Table 1:** Baseline characteristics of participants and comparison by study arm.

Variables			Total (N=25)	Control (n=14)	Intervention (n=11)	*P*
**Sociodemographics**						
	Age (years), mean (SD)		71.7 (11.2)	74.4 (10.4)	68.4 (11.8)	.19
	Gender (male), n (%)		16 (64)	9 (64)	7 (64)	.97
	Race (white), n (%)		22 (88)	13 (93)	9 (82)	.40
	Married, n (%)		11 (44)	7 (50)	4 (36)	.50
	Lives alone, n (%)		10 (40)	6 (43)	4 (36)	.74
	Education (≥1 years of college), n (%)		18 (72)	11 (79)	7 (63)	.41
**Technology use and attitudes**						
	Internet user, n (%)		16 (64)	10 (71)	6 (55)	.38
	**Mobile phone ownership, n (%)**					
		No mobile phone	3 (7)	1 (7)	2 (18)	.69
		Cellphone	18 (72)	11 (79)	7 (64)	
		Smartphone	4 (16)	2 (14)	2 (18)	
**Medication adherence**						
	MOS-Adhere (adherent), n (%)		18 (72)	11 (79)	7 (64)	.41
	Using a medication reminder tool, n (%)		18 (72)	11 (79)	7 (64)	.41
**Health status**						
	**NYHA Functional Classification, n (%)**					
		Class I	12/24 (50)	11/13 (85)	1 (9)	.001
		Class II	10/24 (42)	1/13 (8)	9 (82)	
		Class III	2/24 (8)	1/13 (8)	1 (9)	
		Class IV				
	**Self-rated health, n (%)**					
		Poor	1 (4)	0 (0)	1 (9)	.68
		Fair	11 (44)	6 (43)	5 (46)	
		Good	10 (40)	5 (36)	5 (46)	
		Very good	2 (8)	2 (14)	0 (0)	
		Excellent	1 (4)	1 (7)	0 (0)	
	Depression (PHQ-8), mean (SD)		5.0 (4.8)	3.5 (4.6)	6.8 (4.5)	.08
	**Health-related quality of life (MLHFQ), mean (SD)^a^**					
		MLHFQ total score	34.3 (25.6)	26.2 (23.1)	43.7 (25.9)	.10
		MLHFQ physical score	15.7 (12.7)	10.8 (11.4)	21.9 (11.8)	.03
		MLHFQ emotional score	6.5 (6.7)	5.9 (6.2)	7.2 (7.7)	.65

^a^The MLHFQ is scored so that higher values indicate an adverse impact on quality of life.

At baseline, intervention participants appeared to have been in significantly worse health than participants assigned to the control arm. Intervention participants had more severe HF symptoms, as assessed using the NYHA Functional Classification. In the intervention arm, 9% (1/11) were Class I and 82% (9/11) were Class II compared to controls, which were 85% (11/13) Class I and 8% (1/13) Class II (*P*=.001). The MLHFQ physical subscale was significantly worse in the intervention group compared to controls (mean 21.9, SD 11.8 vs mean 10.8, SD 11.4, *P*=.03). There were no other statistically significant differences in baseline characteristics.

### Emergency Department Visits and Hospitalizations

Table 2 presents the number of participants who had one or more ED visits or hospitalizations. There was no statistically significant difference between study arms in ED visits or for HF-related and non-HF-related hospitalizations. There were significantly fewer all-cause hospitalizations among the intervention group compared to controls. Approximately 9% (1/11) of intervention participants were hospitalized one or more times compared to 50% (7/14) of control participants (*P=*.04), a relative risk reduction in hospitalization of approximately 82%. Additional analysis using logistic regression was conducted to control for baseline differences in NYHA classification and the MLHFQ physical subscale. However, the addition of these baseline measures did not improve the fit of the model and were not significant.

**Table 2:** Emergency department visits and hospitalizations (one or more occurrences during the 90-day study period) by study arm.

Variables		Control, n (%) (n=14)	Intervention, n (%) (n=11)	*P*
**Emergency department visits**				
	Heart failure-related	3 (21)	1 (9)	.60
	Non-heart failure-related	4 (29)	3 (27)	>.99
	All cause^a^	6 (43)	3 (27)	.68
**Hospitalizations**				
	Heart failure-related	4 (29)	1 (9)	.34
	Non-heart failure-related	4 (29)	1 (9)	.34
	All cause^a^	7 (50)	1 (9)	.04

^a^Some participants reported both HF- and non-HF-related ED visit or hospitalization; therefore, the percentage of participants with one or more all-cause ED visits or hospitalizations is lower than the sum of the two types.

Table 3 presents the results comparing the total number of ED visits, hospitalizations, and length of stay if admitted to the hospital. The number of ED and hospitalization visits did not differ significantly between study arms. The intervention arm had significantly fewer all-cause hospitalization days compared to the controls (4 vs 34, *P*=.03) and there was a reduction in the number of days for HF-related and non-HF-related hospitalizations, but this did not meet statistical significance (*P*=.24).

**Table 3:** Emergency department visits, hospitalizations, and hospitalization length of stay for both study arms at closeout.

Variables		Control (n=14)		Intervention (n=11)		*P*
		n	Mean (SD)	n	Mean (SD)	
**Emergency department visits**						
	All cause	7	0.50 (0.65)	4	0.36 (0.67)	.50
	Heart failure	3	0.21 (0.43)	1	0.09 (0.30)	.41
	Non-heart failure	4	0.29 (0.47)	3	0.27 (0.46)	.94
**Hospitalizations**						
	All cause	8	0.57 (0.65)	2	0.18 (0.60)	.06
	Heart failure	4	0.29 (0.47)	1	0.09 (0.30)	.24
	Non-heart failure	4	0.29 (0.47)	1	0.09 (0.30)	.24
**Hospitalization length of stay (days)**						
	All cause	34	2.43 (3.13)	4	0.36 (1.21)	.03
	Heart failure	19	1.36 (2.68)	2	0.18 (0.60)	.20
	Non-heart failure	15	1.07 (2.06)	2	0.18 (0.60)	.21

### Medication Adherence

Table 4 presents the results of tests for differences between the intervention and control arms on medication adherence, health status, and HRQoL. At study closeout, there was no significant difference in self-reported medication adherence. The MOS single-item measure indicated that 67% of participants in the two study arms were adherent. Objective data on adherence generated by the MedSentry device used in the intervention group indicated monthly adherence levels of 94% to 99% after efforts made by the monitoring center staff to confirm medications were taken as prescribed.

**Table 4:** Medication adherence, health status, and health-related quality of life for both study arms at closeout.^a^

Variables			Control (n=13)	Intervention (n=11)	*P*
**Medication adherence**					
	MOS-Adhere (adherent), n (%)		9 (69)	8 (73)	.61
	**Adherence, MedSentry device data, mean % (SD)**				
		Total (90 days)		96.8 (7.2)	
		Month 1 (days 1-30)		98.7 (2.1)	
		Month 2 (days 31-60)		97.4 (5.0)	
		Month 3 (days 61-90)		94.2 (11.2)	
**Health status**				
	**Self-rated health**				
		Poor	0/12 (0)	1 (9)	.82
		Fair	5/12 (42)	3 (27)	
		Good	6/12 (50)	6 (55)	
		Very Good	1/12 (8)	1 (9)	
		Excellent	0/12 (0)	0 (0)	
	Depression (PHQ-8), mean (SD)		3.5 (3.6)	4.5 (2.6)	.46
	**Health-related quality of life (MLHFQ), mean (SD)^b^**				
		MLHFQ total score	28.2 (22.3)	62.2 (20.6)	.002
		MLHFQ physical score	11.2 (10.8)	29.8 (10.7)	.001
		MLHFQ emotional score	5.6 (6.0)	11.5 (6.7)	.03

^a^Missing cases for some comparisons is because of incomplete responses on the closeout questionnaire.

^b^The MLHFQ is scored so that higher values indicate an adverse impact on quality of life.

### Health Status and Health-Related Quality of Life

There was no significant difference in self-rated health or in depression assessed using the PHQ-8. However, the intervention arm had significantly worse HRQoL as measured using the MLHFQ and on the two subscales that assessed physical and emotional dimensions. For example, intervention arm participants had a mean MLHFQ score of 62.2 (SD 20.6) and the control arm had a mean of 28.2 (SD 22.3, *P*=.002).

### Usefulness and Satisfaction

A series of questions on the closeout questionnaire were used to assess the intervention participants’ ratings of the usefulness of 10 MedSentry medication monitoring system features (see Table 5). At least 50% of participants ranked all 10 features as “mostly” or “extremely” useful. The features rated most useful (based on number of “extremely” useful ratings) were the scan that ensured the device was loaded correctly, receiving a call if the wrong medicine was taken, and lights indicating which bin to open. The two features participants rated as least useful were the medication adherence reports sent to the participants’ physician and caregiver and the arrows on the device control panel. Two questions were used to assess whether participants would recommend or want to continue to use the MedSentry medication monitoring system if it were available (results not shown); 70% (7/10) strongly agreed that they would recommend the MedSentry medication monitoring system to a friend or family member and 50% (5/10) strongly agreed they would like to continue to use the MedSentry medication monitoring system if it was made available.

**Table 5:** Usefulness of MedSentry medication monitoring system features (n=11).^a^

Usefulness items	Did not use	Not at all	A little	Mostly	Extremely
Scan to ensure meds loaded correctly	0 (0)	1 (9)	1 (9)	1 (9)	8 (73)
Receiving a call if removed wrong meds	1 (9)	0 (0)	2 (18)	1 (9)	7 (64)
Glowing bins for meds to take	0 (0)	0 (0)	3 (27)	1 (9)	7 (64)
Alarm if wrong bin opened	1 (9)	0 (0)	1 (9)	3 (27)	6 (55)
Receiving a call if missed meds	0 (0)	1 (9)	2 (18)	2 (18)	6 (55)
Ring tone reminder to take meds	0 (0)	0 (0)	2 (18)	3 (27)	6 (55)
Reminder messages on control panel	1 (9)	2 (18)	1 (9)	2 (18)	5 (46)
Call button to request a call	1 (9)	1 (9)	2 (18)	3 (27)	4 (36)
Reports sent to doctor and caregiver	4 (36)	0 (0)	0 (0)	3 (27)	4 (36)
The arrows on the control panel (n=6)	3 (50)	0 (0)	0 (0)	1 (17)	2 (33)

^a^Missing cases for some comparisons was because of incomplete responses on the closeout questionnaire. Percentages may not total to 100 due to rounding error.

## Discussion

This study explored the effects of the MedSentry medication monitoring system, a remote medication management system among HF patients. We hypothesized that remote medication monitoring would be associated with fewer hospitalizations and ED visits, increased medication adherence, and improved HRQoL compared to usual care. We further sought to assess user satisfaction and usability among participants in the intervention arm.

The first hypothesis was that the MedSentry medication monitoring system would be associated with fewer hospitalizations and ED visits compared to usual care. There were a significantly lower number of all-cause hospitalizations in the intervention arm relative to the controls. In the intervention arm, approximately 9% (1/11) were hospitalized one or more times compared to 50% (7/14) in the control arm, a reduction in the relative risk of approximately 82%. Furthermore, there were a significantly lower number of all-cause days hospitalized in the intervention arm compared to controls and fewer HF and non-HF days hospitalized in the intervention arm compared to controls, although this difference did not reach statistical significance. However, there was no statistically significant difference in the number of ED visits.

The reduction in the days hospitalized is encouraging given the small sample size of this pilot study and the relatively poorer health of the intervention participants compared to the controls. Previous studies examining the effects of HF telemonitoring interventions have been mixed. A recent large scale RCT found no significant reduction in hospitalization and ED visits [16], whereas systematic reviews [15,17] and smaller studies have found positive effects [19,22-26]. This variation is likely due to differences in the type of telemonitoring intervention, inclusion criteria and variations in the health of patients enrolled in the programs, and real-world differences in implementation of research studies across multiple clinics and sites [9]. A recent analysis of a standard-of-care HF telemonitoring program, combined with telephone nursing support, was found to be associated with a reduction in hospitalization rates and mortality up to 120 days postdischarge compared to controls, which marked the end of the telemonitoring program [18]. However, 8 months later there was no difference between those who participated in the program and matched controls. Thus, there is reason to believe that telemonitoring systems can substantially reduce health care utilization following HF-related hospitalization if systems are well integrated with existing care delivery and targeted toward patients at risk. Additionally, existing telemonitoring systems could be complemented by other interventions strategies to extend telemonitoring beyond 90 to 120 days, which is typical of most programs.

Objectively measured medication adherence in the intervention group ranged from 94% to 99%, as measured by data generated directly by the devices. However, the intervention did not improve medication adherence as measured by self-report (ie, MOS). The observed lack of improvement may be due to the lack of reliability in using self-reports as a measure of true adherence. For example, in a previous study, researchers found similar self-reported adherence rates (ie, 72%) but objectively measured adherence as low as 54% when comparing the single-item MOS adherence measure to objective measurements using a medication event monitoring system (MEMS) among HF patients [28]. Thus, it is possible that participants overestimated their level of adherence at baseline and may have underreported adherence rates at closeout because they were alerted each time they erred in taking or refilling their medication. In fact, over the course of the study, the monitoring center detected weekly refill errors of 62%, which required calls to participants to correct. Second, at baseline, 72% of patients were using some form of medication reminder system and all participants had completed a telemonitoring intervention.

In our review of the literature, we were unable to find studies evaluating telemonitoring interventions to improve medication adherence for HF patients or patients with complex medication regimens. However, a meta-analysis of other types of medication adherence interventions for HF patients’ found, overall, that programs have a modest effect, especially when the focus is on patients and their medication-taking behaviors [32]. This meta-analysis also found that a focus on interventions aimed at modifying one patient behavior are more effective in increasing adherence than interventions addressing multiple behaviors. This suggests that innovative telemedicine medication adherence interventions may prove especially effective due to the small number of new behaviors (ie, refilling the device) required by patients and the support provided by the monitoring center staff to guide patients in correcting refill errors, which were found to be considerable in number.

The third hypothesis was that the MedSentry medication monitoring system would be associated with improved HRQoL. In contrast, we found the opposite effect—participants in the intervention arm had a significantly poorer HRQoL at closeout than the control arm as measured using the MLHFQ physical subscale. However, this deterioration in HRQoL might be due to differences in HF status between the two study arms at enrollment. Participants in the intervention arm had significantly poorer HF-related health, as classified by the NYHA Functional Classification and the MLHFQ physical subscale. Thus, intervention arm participants appear to have more severe HF than the control arm participants and this may have contributed to a greater decline in health and HRQoL compared to control arm participants over the course of the study.

Finally, this study assessed usability and patient satisfaction related to using the MedSentry medication monitoring system. Users rated the device highly, with at least half of all participants ranking each listed feature as “mostly” or “extremely” useful, and a majority strongly agreeing they would recommend the MedSentry medication monitoring system to a friend or family member. Patients liked the features of the device that promoted supervised engagement, such as the light indicating which bin to open and the scan to ensure correct loading. Although users liked the idea of receiving a call if they took the wrong medication, they were less amenable to the idea of adherence reports being sent to their doctor and caregiver.

### Limitations

Despite the strengths of using a randomized controlled study design to evaluate the effect of the intervention, there are limitations to this study that should be considered when interpreting results. First, the size of the sample is small and the power to detect differences at baseline and closeout is low. Unfortunately, recruitment was slow and the study was ended early before achieving the original goal of 35 participants per study arm. Future research is needed with a larger sample size to ensure statistical power to evaluate primary and secondary outcomes. One reason many patients declined to participate, or were not approved by physicians, was due to a belief they were managing their medications adequately. Research shows this is often not the case and efforts to further educate providers and patients about this fact may improve enrollment rates.

A second limitation is that self-reported measures of medication adherence are not the best method to assess the effect of remote medication management systems on adherence. When possible, a study design using a MEMS to objectively track medication adherence in the control arm would be preferable to self-reports. Unfortunately, this is difficult to accomplish because no devices exist that make it easy to track the multiple medications that most HF patients have been prescribed. One alternative might be to use a “dummy” device that is similar in design to the test device, but lacks the reminders and alerts and is used only for tracking medication adherence.

Finally, a third limitation is that participants in our two study arms differed significantly in NYHA classification and HRQoL. We found that controlling for these differences using logistic regression models did not change our results. However, future research using a larger sample and/or inclusion/exclusion criteria that is more narrowly defined regarding HF condition at enrollment would be useful in creating a sample that is better balanced on baseline characteristics.

### Conclusion

The MedSentry medication monitoring system is a promising technology that merits continued development and evaluation. Most existing telemedicine HF interventions monitor vital signs and self-reported symptoms for 30 to 90 days postdischarge, whereas the MedSentry medication monitoring system provides a relatively low-cost means to remotely monitor HF patients’ medication adherence. The home device is used to remind patients when they should take their medications and the innovative use of cameras to monitor the contents of each medication bin enables advisors at the remote monitoring center to follow-up with patients by phone if there are missed medications or refill errors. As a standalone system, we found that the use of the MedSenty medication monitoring system was associated with an 80% reduction in the risk of unplanned all-cause hospitalization and a significant decrease in the number of unplanned all-cause days hospitalized in the intervention arm compared to usual care. The MedSentry medication monitoring system may be useful both as a standalone system for patients with complex medication regimens or used to complement existing HF telemonitoring interventions. Telemonitoring medication adherence technologies, such as the MedSentry medication monitoring system, are a promising method to improve the quality of patient care and reduce health care utilization and expenditure for patients with HF and other chronic diseases that require complex medication regimens.

## Multimedia Appendix 1

Enrollment and Closeout Questionnaires.

## Abbreviations

BWH: Brigham Women’s Hospital
ED: emergency department
HF: heart failure
HRQoL: health-related quality of life
MEMS: medication event monitoring system
MGH: Massachusetts General Hospital
MLHFQ: Minnesota Living with Heart Failure Questionnaire
MOS: Medical Outcomes Survey
NYHA: New York Heart Association
PHQ: Patient Health Questionnaire
RCT: randomized controlled trial
