# Corrigendum and Editorial Warning Regarding Use of the MMAS-8 Scale (A Remote Medication Monitoring System for Chronic Heart Failure Patients to Reduce Readmissions: A Two-Arm Randomized Pilot Study)

**DOI:** 10.2196/13125

**Published:** 2019-02-05

**Authors:** Timothy M Hale, Kamal Jethwani, Manjinder Singh Kandola, Fidencio Saldana, Joseph C Kvedar

**Affiliations:** 1 Partners Healthcare Connected Health Boston, MA United States; 2 Massachusetts General Hospital Boston, MA United States; 3 Harvard Medical School Boston, MA United States; 4 Brigham & Women's Hospital Internal Medicine Boston, MA United States

## Authors’ Corrigendum

The authors of “A Remote Medication Monitoring System for Chronic Heart Failure Patients to Reduce Readmissions: A Two-Arm Randomized Pilot Study” (JMIR 2016;18(4):e91) have made the following changes to the text, tables, references, and supplemental files.

These changes reflect replacing the Morisky Medication Adherence Scale with the Medical Outcomes Study adherence measure. As the outcomes from the two measures are similar, this change will not substantially impact the interpretation of the findings and conclusions for this study.

1. In the “Data Collection and Outcome Measures” subsection of the Methods:
  - In the second paragraph, the sentence “Medication adherence was assessed using two self-reported measures” has been changed to “Medication adherence was assessed using a self-reported measure and data collected by the device.”
  - In the second paragraph, the sentence “The 8-item Morisky Medication Adherence Scale (MMAS) is a valid and reliable instrument that is scored from 0 to 8 to yield three categories of adherence: 0=high adherence, 1-2=medium adherence, and ≥3=low adherence [43]” has been removed. Because the only in-text mention of reference 43 has been removed, all subsequent references have been renumbered in the text and reference list accordingly.
  - In the second paragraph, the sentence “The second measure was a single question from the Medical Outcomes Study (MOS) [44,45]” has been changed to “Self-reported adherence was assessed using a single question from the Medical Outcomes Study (MOS) [43,44].”
2. In the “Baseline Characteristics” subsection of the Results:
  - In the first paragraph, the sentence “Medication adherence was low (MMAS ≥3) for 27% (6/22) and medium to high (MMAS 0-2) for 73% (16/22)” has been changed to “Seventy-two percent (18/25) of participants were categorized as ‘adherent’ based on self-reported adherence.”
  - In Table 1:
    - Under **Sociodemographics**, on the line “Age (years), mean (SD)”, the superscripted “a” has been removed.
    - Under **Medication Adherence**, the Morisky MMAS-8 scale and corresponding subrows have been removed.
    - On the line “**Health-related quality of life (MLHFQ), mean (SD)**^b^”, the superscripted “b” has been changed to “a”.
    - The original footnote “a” (“The MMAS is scored so that higher values indicate low adherence”) has been removed, and the original footnote “b” (“The MLHFQ is scored so that higher values indicate an adverse impact on quality of life”) has been relabeled “a”.
3. In the “Medication Adherence” subsection of the Results:
  - In the first paragraph, the sentence “Assessed using the MMAS, 75% (9/12) of the control group and 60% (6/10) of the intervention group were categorized as adherent (MMAS score=0-2)” has been removed.
  - In Table 4:
    - Under **Medication Adherence**, the Morisky MMAS-8 scale and corresponding subrows have been removed.
    - On the line “MOS-Adhere (adherent), n (%)”, “8 (62)” has been changed to “9 (69)”, and “.56” has been changed to “.61”.
    - On the line “**Health-related quality of life (MLHFQ), mean (SD)**^c^”, the superscripted “c” has been changed to “b”.
    - The original footnote “b” (“The MMAS is scored so that higher values indicate low adherence”) has been removed, and the original footnote “c” (“The MLHFQ is scored so that higher values indicate an adverse impact on quality of life”) has been relabeled “b”.
4. In the Discussion section, the sentence, “However, based on data from our primary method of assessing change in medication adherence (self-reported MMAS), the intervention did not appear to improve medication adherence” has been changed to “However, the intervention did not improve medication adherence as measured by self-report (ie, MOS).”
5. “MMAS: Morisky Medication Adherence Scale” has been removed from the Abbreviations list.
6. Multimedia Appendix 1 “Enrollment and Closeout Questionnaires” has been replaced with the new version.

The correction will appear in the online version of the paper on the JMIR website on February 5, 2019, together with the publication of this correction notice. Because this was made after submission to PubMed, PubMed Central, and other full-text repositories, the corrected article also has been resubmitted to those repositories.

## Editorial Notice

Authors and journal had to publish this correction due to legal threats by Steven Trubow and Donald Morisky from the company MMAS Research LLC, the copyright holder of the instrument. This is unfortunately not an isolated case, as the developers of this scale are known to comb the literature and ask those who used the scale for research to pay for a retroactive license which may cost thousands or tens of thousands of dollars, and to add references to their work [1]. This is now the third correction JMIR has to publish related to studies using the MMAS instrument.

The Committee on Publication Ethics (COPE) has recently discussed the ethics of this type of behavior by copyright holders of scales (“holding authors to ransom in this way”) and recommends to emphasize “the fact that this is not good for the advancement of scientific knowledge or in the public interest” [2]. As open access and open science publisher we remind our authors of our policies and preference for public and free availability of research tools, including questionnaires [3]. We actively discourage use of instruments which are not available under a Creative Commons Attribution license, and encourage our authors to use or develop/validate new instruments. We continue with our special call for papers for short paper instruments or electronic tools licensed under Creative Commons or available under an Open Source license that can be used as a free alternative to measure medication adherence, and will waive the article submission fee for such development and validation papers.

## Appendix

Multimedia Appendix 1 Enrollment and Closeout Questionnaires.
