# Supplementary material for: A Remote Medication Monitoring System for Chronic Heart Failure Patients to Reduce Readmissions: A Two-Arm Randomized Pilot Study
Source: J Med Internet Res. 2016 May 6;18(4):e91. doi: 10.2196/jmir.5256 (PMC4890732; doi:10.2196/jmir.5256)
Supplement: Supplementary file 1 [file jmir_v18i5e91_app1.pdf]

MedSentry Enrollment Questionnaire

Number: ________________

Date: ________________

Section A: Demographics

Please answer the following questions about yourself.

1. **What is your age?** __________ *(Write in number of years.)*

2. **What is your gender?**
   (Circle the number of your answer.)

   1 Male

   2 Female

3. **What is your marital status**
   (Circle the number of your answer.)

   1 Married

   2 Living with partner

   3 Divorced or separated

   4 Widowed

   5 Single, never been married

4. **Including yourself, how many adults (ages 18 years and older) live in your household?**

   __________ *(Write in number of adults, including yourself.)*

5. **What is the highest grade at school or years in college that you have completed?**
   (Circle the number of your answer.)

   1 1st – 8th grade

   2 9th – 11th grade

   3 12th grade, completed high school, or GED

   4 1 to 3 years of college

   5 4 or more years of college

6. **Are you Hispanic or Latino?**
   (Circle the number of your answer.)

   1 Yes

   2 No

7. Which one or more of the following would you say is your race?
(Circle the number of your answers, more than one may apply.)

1 American Indian or Alaska Native

2 Asian

3 Black or African American

4 Native Hawaiian or other Pacific Islander

5 White

6 Other, please specify: ______________________________

8. What is your current employment status?
(Circle the number of your answer.)

1 Employed full-time (includes self-employment)

2 Employed part-time (includes self-employment)

3 Unemployed

4 Homemaker

5 Student

6 Retired

7 Disabled

8 Other, please specify: ______________________________

Section B: Social Support

Please answer the following questions about those you can turn to for help and support.

9. How often is each of the following statements true for you?
(Circle the number of your answer.)

		Never	Rarely	Sometimes	Usually	Always
a.	I have someone who will listen to me when I need to talk.	1	2	3	4	5
b.	I have someone to confide in or talk to about myself or my problems.	1	2	3	4	5
c.	I have someone who makes me feel appreciated	1	2	3	4	5
d.	I have someone to talk with when I have a bad day.	1	2	3	4	5
e.	I have someone to give me good advice about a crisis if I need it.	1	2	3	4	5
f.	I have someone to turn to for suggestions about how to deal with a problem.	1	2	3	4	5
g.	I have someone to give me information if I need it.	1	2	3	4	5
h.	I get useful advice about important things in my life.	1	2	3	4	5

10. How often can you turn to someone for help with each of the following?
(Circle the number of your answer.)

		Never	Rarely	Sometimes	Usually	Always
a.	Do you have someone to help you if you are confined to bed?	1	2	3	4	5
b.	Do you have someone to take you to the doctor if you need it?	1	2	3	4	5
c.	Do you have someone to help you with your daily chores if you are sick?	1	2	3	4	5
d.	Do you have someone to run errands if you need it?	1	2	3	4	5

Section B: Your Health

Please answer a few questions about your health.

11. In general, would you say your health is…
(Circle the number of your answer.)

1 Excellent

2 Very good

3 Good

4 Fair

5 Poor

12. Over the last <u>2 weeks,</u> how often have you been bothered by any of the following problems?
(Circle the number of your answer.)

		Not at all	Several days	More than half the days	Nearly every day
a.	Little interest or pleasure in doing things	0	1	2	3
b.	Feeling down, depressed, or hopeless	0	1	2	3
c.	Trouble falling or staying asleep, or sleeping too much	0	1	2	3
d.	Feeling tired or having little energy	0	1	2	3
e.	Poor appetite or overeating	0	1	2	3
f.	Feeling bad about yourself – or that you are a failure or have let yourself or your family down	0	1	2	3
g.	Trouble concentrating on things, such as reading a newspaper or watching television	0	1	2	3
h.	Moving or speaking so slowly that other people could have noticed. Or the opposite – being so fidgety or restless that you have been moving around a lot more than usual	0	1	2	3

The following questions ask how much your heart failure (heart condition) affected your life during the past month (4 weeks). After each question, circle the number (1, 2, 3, 4 or 5) to show how much your life was affected. If a question does not apply to you, circle the number 0 after that question.

13. Did your heart failure prevent you from living as you wanted during the past month (4 weeks) by…

		**No**	**Very Little**				**Very Much**
a.	Causing swelling in your ankles or legs?	0	1	2	3	4	5
b.	Making you sit or lie down to rest during the day?	0	1	2	3	4	5
c.	Making your walking about or climbing stairs difficult?	0	1	2	3	4	5
d.	Making your working around the house or yard difficult?	0	1	2	3	4	5
e.	Making your going places away from home difficult?	0	1	2	3	4	5
f.	Making your sleeping well at night difficult?	0	1	2	3	4	5
g.	Making your relating to or doing things with your friends or family difficult?	0	1	2	3	4	5
h.	Making your working to earn a living difficult?	0	1	2	3	4	5
i.	Making your recreational pastimes, sports, or hobbies difficult?	0	1	2	3	4	5
j.	Making your sexual activities difficult?	0	1	2	3	4	5
k.	Making you eat less of the foods you like?	0	1	2	3	4	5
l.	Making you short of breath?	0	1	2	3	4	5
m.	Making you tired, fatigued or low on energy?	0	1	2	3	4	5
n.	Making you stay in a hospital?	0	1	2	3	4	5
o.	Costing you money for medical care?	0	1	2	3	4	5
p.	Giving you side effects from treatments?	0	1	2	3	4	5
q.	Making you feel you are a burden to your family or friends?	0	1	2	3	4	5
r.	Making you feel a loss of self-control in your life?	0	1	2	3	4	5
s.	Making you worry?	0	1	2	3	4	5
t.	Making it difficult for you to concentrate or remember things?	0	1	2	3	4	5
u.	Making you feel depressed?	0	1	2	3	4	5

Section C: Taking Your Medications

14. Are you currently using a medication reminder tool (such as a 7-day pill box, upside-down bottles, or someone who reminds you to take your medications)?
(Circle the number of your answer.)

1 Yes [if yes, please describe below]

2 No

If yes, please describe: ______________________________

____

15. Please circle ALL of the reasons that you sometimes do not take you medication.
(Circle the number for your answers. You may circle more than one answer.)

1 I forgot

2 I had no symptoms or the symptoms went away

3 I wanted to save money

4 I didn't think the drugs were effective

5 I didn't think I needed to take them

6 I was troubled by the side-effects

7 I had difficulty getting the prescriptions filled

8 I was confused by all the drugs that I had to take

16. How often did you take your medications as prescribed (on time without skipping doses) during the past <u>4 weeks</u>?
(Circle the number of your answer.)

1 None of the time

2 A little of the time

3 Some of the time

4 A good bit of the time

5 Most of the time

6 All of the time

17. People sometimes find it difficult to take their medication as directed by their physician. As directed means consistently taking the amount of medication as prescribed at the time prescribed. Please find the statement below that best describes the way you feel right now about taking your heart failure medication as directed.
(Circle the number of your answer.)

1 No, I do not take my heart failure medication as directed right now, and I am not considering taking my heart failure medication as directed.

2 No, I do not take my heart failure medication as directed right now, but I am considering taking my heart failure medication as directed.

3 No, I do not take my heart failure medication as directed, but I am planning to start taking my heart failure medication as directed.

4 Yes, right now I consistently take my heart failure medication as directed.

↓

18. If you answered "YES" to the question above, how long have you been taking your heart failure medication as directed?

1 3 months or less

2 more than 3 months to 6 months

3 more than 6 months to 12 months

4 more than 12 months

19. How much do you agree or disagree with the following statements?
(Circle the number of your answer.)

		Strongly disagree	Disagree	Uncertain	Agree	Strongly agree
a.	My current health depends on my medicines	1	2	3	4	5
b.	Having to take medicines worries me	1	2	3	4	5
c.	My life would be impossible without my medicines	1	2	3	4	5
d.	I sometimes worry about the long-term effects of my medicines	1	2	3	4	5
e.	Without my medicines, I would be very sick	1	2	3	4	5
f.	My medicines are a mystery to me	1	2	3	4	5
g.	My health in the future will depend on my medicines	1	2	3	4	5
h.	My medicines disrupt my life	1	2	3	4	5
i.	I sometimes worry about becoming too dependent on my medicines	1	2	3	4	5
j.	My medicines protect me from becoming worse	1	2	3	4	5
k.	My medicines give me unpleasant side effects	1	2	3	4	5
l.	Doctors use too many medicines	1	2	3	4	5
m.	People who take medicines should stop their treatment for a while every now and then	1	2	3	4	5
n.	Most medicines are addictive	1	2	3	4	5
o.	Medicines do more harm than good	1	2	3	4	5
p.	All medicines are poisonous	1	2	3	4	5
q.	Doctors place too much trust on medicines	1	2	3	4	5
r.	If doctors had more time with patients they would prescribe fewer medicines	1	2	3	4	5

20. How confident are you that you can take your medicines correctly…
(Circle the number of your answer.)

		Not confident	Somewhat confident	Very confident
a.	When you take several different medicines each day?	1	2	3
b.	When you have a busy day planned?	1	2	3
c.	When you are away from home?	1	2	3
d.	When no one reminds you to take the medicine?	1	2	3
e.	When you take medicines more than once a day?	1	2	3
f.	When the schedule to take the medicine is not convenient?	1	2	3
g.	When your normal routine gets messed up?	1	2	3
h.	When you get a refill of your old medicines and some of the pills look unusual?	1	2	3
i.	When you are not sure how to take the medicine?	1	2	3
j.	When you are not sure what time of day to take your medicine?	1	2	3
k.	When a doctor changes your medicine?	1	2	3
l.	When they cause some side effects?	1	2	3
m.	When you are feeling sick (like having a cold or the flu)?	1	2	3

Section D: Technology Ownership and Use

21. Do you ever go online to access the Internet or World Wide Web, or to send and receive email? *(Circle the number of your answer.)*

1 Yes

2 No [skip to question 24]

22. When you use the Internet, do you access it through…
(*For each item, please circle the number for your answer. You may answer "YES" to more than one question.***)**

	**Yes**	**No**
a. A regular dial-up telephone line.	1	2
b. Broadband such as DSL, cable or FiOS.	1	2
c. A cellular network (e.g., smartphone, 3G/4G)	1	2
d. A wireless network (Wi-Fi)	1	2

23. Have you ever used the Internet to do any of the following things?
(*For each item, please circle the number for your answer.***)**

	**Yes**	**No**
a. Send or receive email.	1	2
b. Send or receive instant messages or chat online.	1	2
c. Upload pictures to share with others.	1	2
e. Look for health or medical information online.	1	2
f. Track weight, diet or exercise routine.	1	2
g. Track any other health indicators like blood pressure, sleep patterns, headaches, etc.	1	2
h. Check your bank account balance or do any online banking.	1	2
i. Use a social networking service like Facebook or MySpace.	1	2

24. Do you have any of the following devices?
(*For each item, please circle the number for your answer.***)**

	**Yes**	**No**
a. A landline telephone.	1	2
b. A desktop computer.	1	2
c. A laptop computer or netbook.	1	2
d. A tablet computer like an iPad.	1	2

25. Do you have a cell phone or Blackberry or iPhone or other device that is also a cell phone? *(Circle the number of your answer.)*

1 Yes

2 No [skip to question 28]

26. Some cell phones are called "smartphones" because of certain features they have. Is your cell phone a smartphone, such as an iPhone, Android, Blackberry or Windows phone? *(Circle the number of your answer.)*

1 Yes

2 No

3 Not sure or don't know

27. Have you ever used your cell phone or smartphone to do any of the following things? **(***For each item, please circle the number for your answer.***)**

	**Yes**	**No**
a. Send or receive email.	1	2
b. Send or receive text messages.	1	2
c. Take a picture to share with others.	1	2
d. Access the Internet.	1	2
e. Look for health or medical information online.	1	2
f. Track weight, diet or exercise routine.	1	2
g. Track any other health indicators like blood pressure, sleep patterns, headaches, etc.	1	2
h. Check your bank account balance or do any online banking.	1	2
i. Use a social networking service like Facebook or MySpace.	1	2

28. How much do you agree or disagree with <u>each</u> of the following statements?
(*For each item, please circle the number for your answer.***)**

	**Strongly agree**	**Somewhat agree**	**Somewhat disagree**	**Strongly disagree**
a. I am willing to try new technology.	1	2	3	4
b. I plan to make greater use of technology in the future to manage my health.	1	2	3	4
c. I generally feel confident using new technology.	1	2	3	4
d. I generally feel confident that I can use new technology to manage my medical condition.	1	2	3	4
e. I worry about security issues of sending health information by the Internet.	1	2	3	4
f. It is easy for me to follow instructions and set up new technology.	1	2	3	4
g. I have no difficulty setting up computers or Internet modems.	1	2	3	4
h. Learning how to use new technology is easy for me.	1	2	3	4
i. New technology can be useful in keeping me healthy.	1	2	3	4
j. I find using new technology to be a waste of time.	1	2	3	4

29. Is there anything else that you would like to tell us? If so, please write this in the space below.

____

____

____

____

____

____

Your answers are important to us. Please take a moment to check that you have answered all of the questions.

Thank you for taking the time to complete this survey!

MedSentry Closeout Questionnaire

Number: ________________

Date: ________________

Section A: Taking Your Medications

1. **Are you currently using a medication reminder tool (such as a 7-day pill box, upside-down bottles, or someone who reminds you to take your medications)?**
(Circle the number of your answer.)

   1 Yes [if yes, please describe below]

   2 No

If yes, please describe: ____

____

2. **How often did you take your medications as prescribed (on time without skipping doses) during the past <u>4 weeks</u>?**
(Circle the number of your answer.)

   1 None of the time

   2 A little of the time

   3 Some of the time

   4 A good bit of the time

   5 Most of the time

   6 All of the time

3. **People sometimes find it difficult to take their medication as directed by their physician. As directed means consistently taking the amount of medication as prescribed at the time prescribed. Please find the statement below that best describes the way you feel right now about taking your heart failure medication as directed.**
(Circle the number of your answer.)

   1 No, I do not take my heart failure medication as directed right now, and I am not considering taking my heart failure medication as directed.

   2 No, I do not take my heart failure medication as directed right now, but I am considering taking my heart failure medication as directed.

   3 No, I do not take my heart failure medication as directed, but I am planning to start taking my heart failure medication as directed.

   4 Yes, right now I consistently take my heart failure medication as directed.

   4. **If you answered "YES" to the question above, how long have you been taking your heart failure medication as directed?**

      1 3 months or less

      2 more than 3 months to 6 months

      3 more than 6 months to 12 months

      4 more than 12 months

5. How much do you agree or disagree with the following statements?
(Circle the number of your answer.)

		Strongly disagree	Disagree	Uncertain	Agree	Strongly agree
a.	My current health depends on my medicines	1	2	3	4	5
b.	Having to take medicines worries me	1	2	3	4	5
c.	My life would be impossible without my medicines	1	2	3	4	5
d.	I sometimes worry about the long-term effects of my medicines	1	2	3	4	5
e.	Without my medicines, I would be very sick	1	2	3	4	5
f.	My medicines are a mystery to me	1	2	3	4	5
g.	My health in the future will depend on my medicines	1	2	3	4	5
h.	My medicines disrupt my life	1	2	3	4	5
i.	I sometimes worry about becoming too dependent on my medicines	1	2	3	4	5
j.	My medicines protect me from becoming worse	1	2	3	4	5
k.	My medicines give me unpleasant side effects	1	2	3	4	5
l.	Doctors use too many medicines	1	2	3	4	5
m.	People how take medicines should stop their treatment for a while every now and then	1	2	3	4	5
n.	Most medicines are addictive	1	2	3	4	5
o.	Medicines do more harm than good	1	2	3	4	5
p.	All medicines are poisonous	1	2	3	4	5
q.	Doctors place too much trust on medicines	1	2	3	4	5
r.	If doctors had more time with patients they would prescribe fewer medicines	1	2	3	4	5

6. How confident are you that you can take your medicines correctly…
(Circle the number of your answer.)

		Not confident	Somewhat confident	Very confident
a.	When you take several different medicines each day?	1	2	3
b.	When you have a busy day planned?	1	2	3
c.	When you are away from home?	1	2	3
d.	When no one reminds you to take the medicine?	1	2	3
e.	When you take medicines more than once a day?	1	2	3
f.	When the schedule to take the medicine is not convenient?	1	2	3
g.	When your normal routine gets messed up?	1	2	3
h.	When you get a refill of your old medicines and some of the pills look unusual?	1	2	3
i.	When you are not sure how to take the medicine?	1	2	3
j.	When you are not sure what time of day to take your medicine?	1	2	3
k.	When a doctor changes your medicine?	1	2	3
l.	When they cause some side effects?	1	2	3
m.	When you are feeling sick (like having a cold or the flu)?	1	2	3

Section B: Your Health

Please answer a few questions about your health.

7. In general, would you say your health is…
(Circle the number of your answer.)

1 Excellent
2 Very good
3 Good
4 Fair
5 Poor

8. Compared to 4 months ago, how would you rate your health in general now?
(Circle the number of your answer.)

1 Much better than 4 months ago

2 Somewhat better than 4 months ago

3 About the same as 4 months ago

4 Somewhat worse than 4 months ago

5 Much worse than 4 months ago

9. Over the last <u>2 weeks,</u> how often have you been bothered by any of the following problems?
(Circle the number of your answer.)

		Not at all	Several days	More than half the days	Nearly every day
a.	Little interest or pleasure in doing things	0	1	2	3
b.	Feeling down, depressed, or hopeless	0	1	2	3
c.	Trouble falling or staying asleep, or sleeping too much	0	1	2	3
d.	Feeling tired or having little energy	0	1	2	3
e.	Poor appetite or overeating	0	1	2	3
f.	Feeling bad about yourself – or that you are a failure or have let yourself or your family down	0	1	2	3
g.	Trouble concentrating on things, such as reading a newspaper or watching television	0	1	2	3
h.	Moving or speaking so slowly that other people could have noticed. Or the opposite – being so fidgety or restless that you have been moving around a lot more than usual	0	1	2	3

The following questions ask how much your heart failure (heart condition) affected your life during the past month (4 weeks). After each question, circle the number (1, 2, 3, 4 or 5) to show how much your life was affected. If a question does not apply to you, circle the number 0 after that question.

10. Did your heart failure prevent you from living as you wanted during the past month (4 weeks) by…

		**No**	**Very Little**				**Very Much**
a.	Causing swelling in your ankles or legs?	0	1	2	3	4	5
b.	Making you sit or lie down to rest during the day?	0	1	2	3	4	5
c.	Making your walking about or climbing stairs difficult?	0	1	2	3	4	5
d.	Making your working around the house or yard difficult?	0	1	2	3	4	5
e.	Making your going places away from home difficult?	0	1	2	3	4	5
f.	Making your sleeping well at night difficult?	0	1	2	3	4	5
g.	Making your relating to or doing things with your friends or family difficult?	0	1	2	3	4	5
h.	Making your working to earn a living difficult?	0	1	2	3	4	5
i.	Making your recreational pastimes, sports, or hobbies difficult?	0	1	2	3	4	5
j.	Making your sexual activities difficult?	0	1	2	3	4	5
k.	Making you eat less of the foods you like?	0	1	2	3	4	5
l.	Making you short of breath?	0	1	2	3	4	5
m.	Making you tired, fatigued or low on energy?	0	1	2	3	4	5
n.	Making you stay in a hospital?	0	1	2	3	4	5
o.	Costing you money for medical care?	0	1	2	3	4	5
p.	Giving you side effects from treatments?	0	1	2	3	4	5
q.	Making you feel you are a burden to your family or friends?	0	1	2	3	4	5
r.	Making you feel a loss of self-control in your life?	0	1	2	3	4	5
s.	Making you worry?	0	1	2	3	4	5
t.	Making it difficult for you to concentrate or remember things?	0	1	2	3	4	5
u.	Making you feel depressed?	0	1	2	3	4	5

Section C: Self-Reported Re-Admissions

11. **Were you admitted at any hospital or did you visit the emergency department during the 4-month study period?**
    (Circle the number of your answer.)

    1 Yes

    2 No [if no, skip to question 18]

12. **What type of re-admission was this?**
    (Circle the number of your answer.)

    1 Planned

    2 Urgent care (medical care needed for an immediate, but not life-threatening, health problem)

    3 Emergency (immediate, emergency care for a serious medical condition)

13. **Who referred you for this readmission or emergency department visit?**
    (Circle the number of your answer.)

    1 Primary care physician

    2 Cardiologist

    3 Nurse practitioner

    4 Other, please describe: ________________

14. **What complaints did you have? Please describe:** ____________________________________

____

____

15. **Name of hospital:** _______________________________

16. **Date of admission:** ______________________________

17. **Date if discharge:** _______________________________

Section D: Social Support

Please answer the following questions about those you can turn to for help and support.

18. How often is each of the following statement true for you?
(Circle the number of your answer.)

		Never	Rarely	Sometimes	Usually	Always
a.	I have someone who will listen to me when I need to talk.	1	2	3	4	5
b.	I have someone to confide in or talk to about myself or my problems.	1	2	3	4	5
c.	I have someone who makes me feel appreciated	1	2	3	4	5
d.	I have someone to talk with when I have a bad day.	1	2	3	4	5
e.	I have someone to give me good advice about a crisis if I need it.	1	2	3	4	5
f.	I have someone to turn to for suggestions about how to deal with a problem.	1	2	3	4	5
g.	I have someone to give me information if I need it.	1	2	3	4	5
h.	I get useful advice about important things in my life.	1	2	3	4	5

19. How often can you turn to someone for help with each of the following?
(Circle the number of your answer.)

		Never	Rarely	Sometimes	Usually	Always
a.	Do you have someone to help you if you are confined to bed?	1	2	3	4	5
b.	Do you have someone to take you to the doctor if you need it?	1	2	3	4	5
c.	Do you have someone to help you with your daily chores if you are sick?	1	2	3	4	5
d.	Do you have someone to run errands if you need it?	1	2	3	4	5

Section E: Your Experiences Using the MedSentry Pillbox

The following questions ask about the usefulness of various MedSentry pillbox features. After each question, circle the number (1 = “Not Useful at All” to 4 = “Extremely Useful”) that best represents how useful you found each feature. If you did not use a feature, circle the number 0 for “I Did Not Use It” after that question.

20. How useful are the following features?
(Circle the number of your answer.)

		I did not use it	Not at all useful	A little useful	Mostly useful	Extremely useful
a.	Having the MedSentry pillbox glow to show which pill to take.	0	1	2	3	4
b.	Hearing the ring tone to remind me to take my medication.	0	1	2	3	4
c.	Seeing messages on the color touch-screen reminding me to take my medication.	0	1	2	3	4
d.	Receiving a call after I missed taking my medication.	0	1	2	3	4
e.	The Call Button which allowed me to request a call back from an MedSentry Advisor to talk about the MedSentry pillbox.	0	1	2	3	4
f.	The up and down arrows which allowed me to control the volume and other functions on the MedSentry pillbox.	0	1	2	3	4
g.	Having my medications scanned to make sure I loaded my MedSentry pillbox correctly.	0	1	2	3	4
h.	Being contacted if I removed the wrong medication from the MedSentry pillbox.	0	1	2	3	4
i.	The Weekly Medication Adherence Reports being sent to my treatment provider/caregiver.	0	1	2	3	4
k.	Having the alarm sound if I opened the wrong medication box or bin.	0	1	2	3	4

21. Did you ever contact, or attempt to contact, technical support for the MedSentry pillbox?
(Circle the number of your answer.)

1 Yes

2 No [if no, skip to question 24]

22. How satisfied were you with the technical support you received?
(Circle the number of your answer.)

1 Very dissatisfied

2 Dissatisfied

3 Satisfied

4 Very satisfied

23. How satisfied were you with the technical support you received when refilling the MedSentry pillbox?
(Circle the number of your answer.)

1 Very dissatisfied

2 Dissatisfied

3 Satisfied

4 Very satisfied

5 Did not use this type of technical support

24. How much do you agree or disagree with the following statements?
(Circle the number of your answer.)

		Strongly Disagree	Disagree	Agree	Strongly Agree
a.	I would recommend the MedSentry pillbox to a friend or family member.	1	2	3	4
b.	If given the option, I would like to continue to use the MedSentry pillbox.	1	2	3	4
c.	The MedSentry pillbox has helped me take my medications more regularly.	1	2	3	4
d.	The MedSentry pillbox has helped reduce the chances of my taking the wrong medication.	1	2	3	4

25. Is there anything else that you would like to tell us? If so, please write this in the space below.

____

____

____

____

____

____

Your answers are important to us. Please take a moment
to check that you have answered all of the questions.

Thank you for taking the time to complete this survey!

MedSentry Closeout Questionnaire

Number: ________________

Date: ________________

Section A: Taking Your Medications

1. Are you currently using a medication reminder tool (such as a 7-day pill box, upside-down bottles, or someone who reminds you to take your medications)?
(Circle the number of your answer.)

1 Yes [if yes, please describe below]

2 No

If yes, please describe: ____

____

2. How often did you take your medications as prescribed (on time without skipping doses) during the past <u>4 weeks</u>?
(Circle the number of your answer.)

1 None of the time

2 A little of the time

3 Some of the time

4 A good bit of the time

5 Most of the time

6 All of the time

3. People sometimes find it difficult to take their medication as directed by their physician. As directed means consistently taking the amount of medication as prescribed at the time prescribed. Please find the statement below that best describes the way you feel right now about taking your heart failure medication as directed.
(Circle the number of your answer.)

1 No, I do not take my heart failure medication as directed right now, and I am not considering taking my heart failure medication as directed.

2 No, I do not take my heart failure medication as directed right now, but I am considering taking my heart failure medication as directed.

3 No, I do not take my heart failure medication as directed, but I am planning to start taking my heart failure medication as directed.

4 Yes, right now I consistently take my heart failure medication as directed.

4. If you answered "YES" to the question above, how long have you been taking your heart failure medication as directed?

1 3 months or less

2 more than 3 months to 6 months

3 more than 6 months to 12 months

4 more than 12 months

5. How much do you agree or disagree with the following statements?
(Circle the number of your answer.)

		Strongly disagree	Disagree	Uncertain	Agree	Strongly agree
a.	My current health depends on my medicines	1	2	3	4	5
b.	Having to take medicines worries me	1	2	3	4	5
c.	My life would be impossible without my medicines	1	2	3	4	5
d.	I sometimes worry about the long-term effects of my medicines	1	2	3	4	5
e.	Without my medicines, I would be very sick	1	2	3	4	5
f.	My medicines are a mystery to me	1	2	3	4	5
g.	My health in the future will depend on my medicines	1	2	3	4	5
h.	My medicines disrupt my life	1	2	3	4	5
i.	I sometimes worry about becoming too dependent on my medicines	1	2	3	4	5
j.	My medicines protect me from becoming worse	1	2	3	4	5
k.	My medicines give me unpleasant side effects	1	2	3	4	5
l.	Doctors use too many medicines	1	2	3	4	5
m.	People how take medicines should stop their treatment for a while every now and then	1	2	3	4	5
n.	Most medicines are addictive	1	2	3	4	5
o.	Medicines do more harm than good	1	2	3	4	5
p.	All medicines are poisonous	1	2	3	4	5
q.	Doctors place too much trust on medicines	1	2	3	4	5
r.	If doctors had more time with patients they would prescribe fewer medicines	1	2	3	4	5

6. How confident are you that you can take your medicines correctly…
(Circle the number of your answer.)

		Not confident	Somewhat confident	Very confident
a.	When you take several different medicines each day?	1	2	3
b.	When you have a busy day planned?	1	2	3
c.	When you are away from home?	1	2	3
d.	When no one reminds you to take the medicine?	1	2	3
e.	When you take medicines more than once a day?	1	2	3
f.	When the schedule to take the medicine is not convenient?	1	2	3
g.	When your normal routine gets messed up?	1	2	3
h.	When you get a refill of your old medicines and some of the pills look unusual?	1	2	3
i.	When you are not sure how to take the medicine?	1	2	3
j.	When you are not sure what time of day to take your medicine?	1	2	3
k.	When a doctor changes your medicine?	1	2	3
l.	When they cause some side effects?	1	2	3
m.	When you are feeling sick (like having a cold or the flu)?	1	2	3

Section B: Your Health

Please answer a few questions about your health.

7. In general, would you say your health is…
(Circle the number of your answer.)

1 Excellent
2 Very good
3 Good
4 Fair
5 Poor

8. Compared to 4 months ago, how would you rate your health in general now?
(Circle the number of your answer.)

1 Much better than 4 months ago
2 Somewhat better than 4 months ago
3 About the same as 4 months ago
4 Somewhat worse than 4 months ago
5 Much worse than 4 months ago

9. Over the last 2 weeks, how often have you been bothered by any of the following problems?
(Circle the number of your answer.)

		Not at all	Several days	More than half the days	Nearly every day
a.	Little interest or pleasure in doing things	0	1	2	3
b.	Feeling down, depressed, or hopeless	0	1	2	3
c.	Trouble falling or staying asleep, or sleeping too much	0	1	2	3
d.	Feeling tired or having little energy	0	1	2	3
e.	Poor appetite or overeating	0	1	2	3
f.	Feeling bad about yourself – or that you are a failure or have let yourself or your family down	0	1	2	3
g.	Trouble concentrating on things, such as reading a newspaper or watching television	0	1	2	3
h.	Moving or speaking so slowly that other people could have noticed. Or the opposite – being so fidgety or restless that you have been moving around a lot more than usual	0	1	2	3

The following questions ask how much your heart failure (heart condition) affected your life during the past month (4 weeks). After each question, circle the number (1, 2, 3, 4 or 5) to show how much your life was affected. If a question does not apply to you, circle the number 0 after that question.

10. Did your heart failure prevent you from living as you wanted during the past month (4 weeks) by…

		**No**	**Very Little**				**Very Much**
a.	Causing swelling in your ankles or legs?	0	1	2	3	4	5
b.	Making you sit or lie down to rest during the day?	0	1	2	3	4	5
c.	Making your walking about or climbing stairs difficult?	0	1	2	3	4	5
d.	Making your working around the house or yard difficult?	0	1	2	3	4	5
e.	Making your going places away from home difficult?	0	1	2	3	4	5
f.	Making your sleeping well at night difficult?	0	1	2	3	4	5
g.	Making your relating to or doing things with your friends or family difficult?	0	1	2	3	4	5
h.	Making your working to earn a living difficult?	0	1	2	3	4	5
i.	Making your recreational pastimes, sports, or hobbies difficult?	0	1	2	3	4	5
j.	Making your sexual activities difficult?	0	1	2	3	4	5
k.	Making you eat less of the foods you like?	0	1	2	3	4	5
l.	Making you short of breath?	0	1	2	3	4	5
m.	Making you tired, fatigued or low on energy?	0	1	2	3	4	5
n.	Making you stay in a hospital?	0	1	2	3	4	5
o.	Costing you money for medical care?	0	1	2	3	4	5
p.	Giving you side effects from treatments?	0	1	2	3	4	5
q.	Making you feel you are a burden to your family or friends?	0	1	2	3	4	5
r.	Making you feel a loss of self-control in your life?	0	1	2	3	4	5
s.	Making you worry?	0	1	2	3	4	5
t.	Making it difficult for you to concentrate or remember things?	0	1	2	3	4	5
u.	Making you feel depressed?	0	1	2	3	4	5

Section C: Self-Reported Re-Admissions

11. Were you admitted at any hospital or did you visit the emergency department during the 4-month study period?
(Circle the number of your answer.)

1 Yes

2 No [if no, skip to question 20]

12. What type of re-admission was this?
(Circle the number of your answer.)

1 Planned

2 Urgent care (medical care needed for an immediate, but not life-threatening, health problem)

3 Emergency (immediate, emergency care for a serious medical condition)

13. Who referred you for this readmission or emergency department visit?
(Circle the number of your answer.)

1 Primary care physician

2 Cardiologist

3 Nurse practitioner

4 Other, please describe: ________________

14. What complaints did you have? Please describe: ____________________________________

____

____

15. Name of hospital: _______________________________

16. Date of admission: ______________________________

17. Date if discharge: _______________________________

Section D: Social Support

Please answer the following questions about those you can turn to for help and support.

18. How often is each of the following statement true for you?
(Circle the number of your answer.)

		Never	Rarely	Sometimes	Usually	Always
a.	I have someone who will listen to me when I need to talk.	1	2	3	4	5
b.	I have someone to confide in or talk to about myself or my problems.	1	2	3	4	5
c.	I have someone who makes me feel appreciated	1	2	3	4	5
d.	I have someone to talk with when I have a bad day.	1	2	3	4	5
e.	I have someone to give me good advice about a crisis if I need it.	1	2	3	4	5
f.	I have someone to turn to for suggestions about how to deal with a problem.	1	2	3	4	5
g.	I have someone to give me information if I need it.	1	2	3	4	5
h.	I get useful advice about important things in my life.	1	2	3	4	5

19. How often can you turn to someone for help with each of the following?
(Circle the number of your answer.)

		Never	Rarely	Sometimes	Usually	Always
a.	Do you have someone to help you if you are confined to bed?	1	2	3	4	5
b.	Do you have someone to take you to the doctor if you need it?	1	2	3	4	5
c.	Do you have someone to help you with your daily chores if you are sick?	1	2	3	4	5
d.	Do you have someone to run errands if you need it?	1	2	3	4	5

20. Is there anything else that you would like to tell us? If so, please write this in the space below.

____

____

____

____

____

____

Your answers are important to us. Please take a moment to check that you have answered all of the questions.

Thank you for taking the time to complete this survey!
